# Postpartum sexual function; the importance of the levator ani muscle

**DOI:** 10.1007/s00192-020-04250-3

**Published:** 2020-02-24

**Authors:** Anne-Marie Roos, Leonie Speksnijder, Anneke B. Steensma

**Affiliations:** 1 Department of Obstetrics and Gynecology, Division of Urogynaecology, Erasmus Medical Centre, Doctor Molewaterplein 40, 3015 GD Rotterdam, The Netherlands; 2 Department of Obstetrics and Gynecology, division of Urogynaecology, Amphia Hospital, Breda, The Netherlands

**Keywords:** Levator ani muscle, Pelvic floor, Postpartum, Sexual dysfunction, Ultrasonography

## Abstract

**Introduction and hypothesis:**

Pelvic floor muscle function plays an important role in female sexual functioning. Smaller genital hiatal dimensions have been associated with sexual dysfunction, mainly dyspareunia. On the other hand, trauma of the levator ani muscle sustained during childbirth is associated with increased genital hiatus, which potentially can affect sexual functioning by causing vaginal laxity. This study aims to determine the association between levator hiatal dimensions and female sexual dysfunction after first vaginal delivery.

**Methods:**

This is a secondary analysis of a prospective observational study. Two hundred four women who had a first, spontaneous vaginal delivery at term between 2012 and 2015 were recruited at a minimum of 6 months postpartum. Thirteen pregnant women were excluded. We analyzed the association of total PISQ-12 score, as well as individual sexual complaints (desire, arousal, orgasm and dyspareunia), with levator hiatal dimensions at rest, with maximum Valsalva and during pelvic floor muscle contraction as measured by 4D transperineal ultrasound. Statistical analysis was performed using linear regression analysis and Mann-Whitney U test.

**Results:**

One hundred ninety-one women were evaluated at a median of 11 months postpartum. There was no significant association between total PISQ-12 score and levator hiatal dimensions. Looking at individual sexual complaints, women with dyspareunia had significantly smaller levator hiatal area and anterior-posterior diameter on maximum Valsalva. By using multivariate logistic regression analysis however we found dyspareunia was not independently associated with levator hiatal dimensions.

**Conclusions:**

After first vaginal delivery sexual dysfunction is not associated with levator hiatal dimensions as measured by 4D transperineal ultrasound.

## Introduction

Childbirth can have a great impact on women's sexual functioning. Postpartum sexual functioning is influenced by both psychological changes associated with the transition into parenthood as well as physical changes such as perineal trauma [1]. Female sexual dysfunction in the DSM V is subdivided into three broad categories, including: sexual interest/arousal disorder, female orgasmic disorder and genitopelvic pain/penetration disorder, including dyspareunia [2]. Previous literature on postpartum sexual function has focused mainly on the complaint of dyspareunia, which is associated with instrumental delivery and the extent of perineal trauma sustained [3].

Physical birth trauma encompasses more than perineal trauma alone. Following first vaginal delivery 13–36% of women sustain trauma of the levator ani muscle [4–7]. This trauma can be divided into levator ani muscle avulsion and levator over-distension or a combination of both. Trauma of the levator ani muscle is associated with increased genital hiatus (levator ballooning) and decreased pelvic floor contractility and is strongly associated with symptoms and clinical signs of prolapse [4, 6–9]. This potentially could affect sexual functioning, as decreased pelvic floor muscle strength has been associated with worse sexual function [10–12].

Literature on the association of genital hiatus, levator trauma and postpartum sexual functioning is sparse. At short-term follow-up levator avulsion was associated with symptoms of vaginal laxity and reduced vaginal sensation, but these complaints did not have appreciable effects on women’s sexual functioning [13, 14]. At 1 year postpartum women with persistent levator avulsion had significantly more bothersome symptoms of a loose vagina. A trend towards a spoiled sex life as a result of these symptoms was found, but did not reach significance [15].

On the other hand, smaller hiatal dimensions have been associated with sexual dysfunction as well, mainly dyspareunia. Women with provoked vestibulodynia (PVD) were shown to have smaller relative increases in hiatal area on maximum Valsalva, which is associated with hypertonia from the pelvic floor muscles [16].

The aim of this study was to determine the nature of pelvic floor muscle involvement in women with sexual dysfunction after first vaginal delivery. We hypothesized that larger levator hiatus and decreased contractibility of the levator ani muscle are associated with female sexual dysfunction, because of the association with symptoms of reduced vaginal sensation and vaginal laxity. We also hypothesized that a small hiatus in rest and decreased relaxation are associated with dyspareunia. For this purpose we evaluated the biometry of the levator hiatus at rest, pelvic floor muscle contraction (PFMC) and maximal Valsalva using 3D/4D transperineal ultrasound in relation to sexual complaints.

## Materials and methods

This is a secondary analysis of a prospective observational study which aimed to evaluate the association of mediolateral episiotomy with levator injury and urogynecological complaints (EpiLeva study) [17]. All participants were recruited at the Amphia Hospital in Breda, The Netherlands, between 2012 and 2015. Women who underwent a first, spontaneous, term vaginal delivery of a singleton in cephalic position were included. Women who delivered prematurely, who delivered a fetus in non-cephalic position, who experienced instrumental vaginal delivery, obstetric anal sphincter injury or cesarean section were not included. The investigators were not involved in the management of the participants’ deliveries.

Women who agreed to participate filled out standardized urogynecological questionnaires, and a detailed examination including 3D/4D transperineal ultrasound was performed at a minimum of 6 months postpartum [17]. Antenatal and intrapartum characteristics were retrospectively obtained from the patients’ medical notes.

### Assessment of sexual function

For assessment of sexual function the Dutch validated version of the Pelvic Organ Prolapse/Urinary Incontinence Sexual Questionnaire-12 (PISQ-12) was used [18]. The PISQ-12 is a 12-item, condition-specific tool for assessing sexual function in women with urinary incontinence and/or pelvic organ prolapse. Responses are measured on a 5-point Likert scale ranging from “never” to “always.” The total score ranges from 0 to 48, with higher scores indicating better sexual function. For analysis of sexual function in this study we analyzed the total PISQ score as well as individual sexual complaints. To define individual complaints we dichotomized PISQ questions. Dyspareunia was defined as answering “always” or “usually” to the question: “Do you feel pain during sexual intercourse?” Reduced desire was defined as answering “seldom” or “never” to the question: “How often do you feel sexual desire?” Inability to reach an orgasm was defined as answering “seldom” or “never” to the question: “Do you climax (have an orgasm) while having sexual intercourse with your partner?” Reduced arousal was defined as answering “seldom” or “never” to the question: “Do you feel sexually excited (turned on) when having sexual activity with your partner?”

### Three/four-dimensional transperineal ultrasound

Three/four-dimensional transperineal ultrasound was performed in supine position and after voiding, using a Voluson E Expert system using a 4–8-MHz RAB abdominal probe (GE Healthcare, Chalfont St. Giles, UK) as previously described by Dietz et al. [19]. The ultrasound volumes were obtained at rest, PFMC and maximal Valsalva. All volumes were obtained by an investigator experienced in performing 3D/4D transperineal ultrasound.

**Fig. 1:**
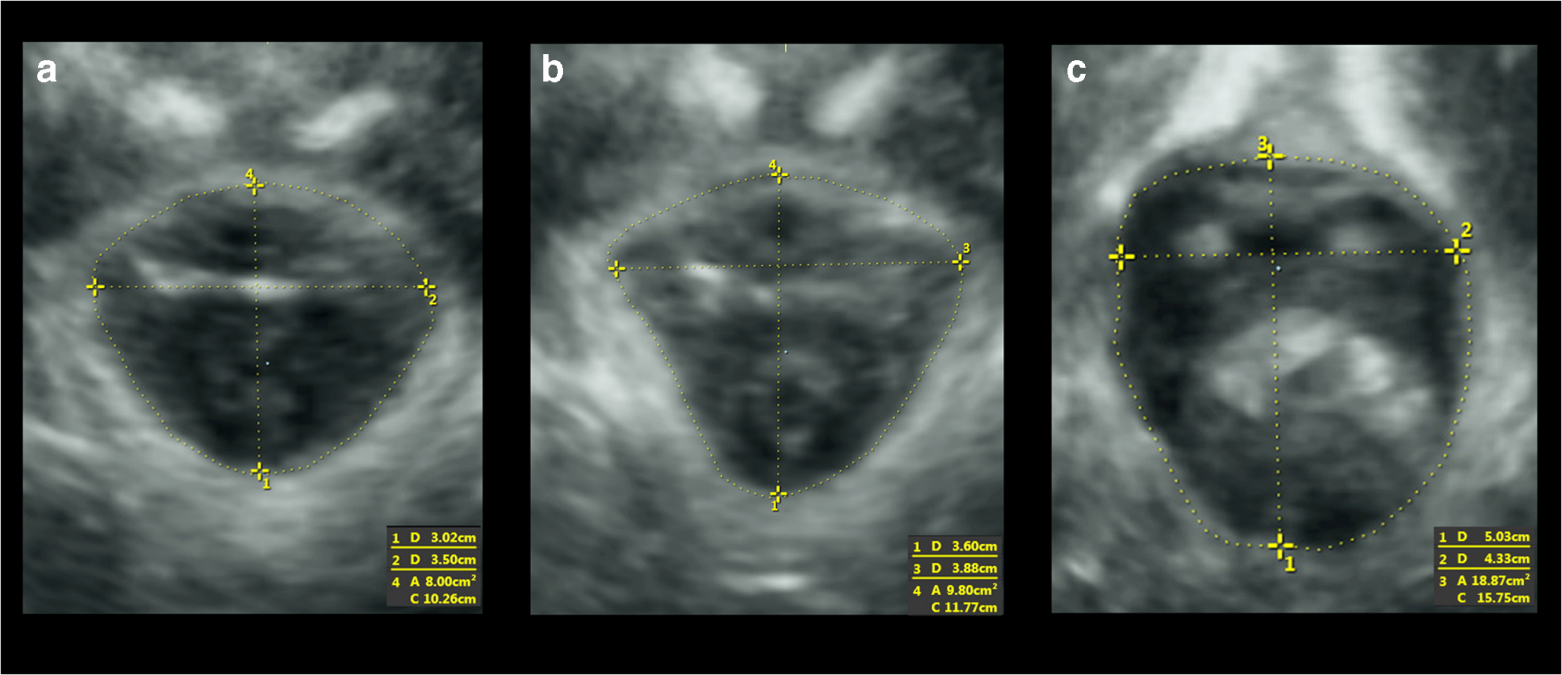
Example of measurement of levator hiatal dimensions in one patient at pelvic floor muscle contraction, at rest and maximal Valsalva

Offline analysis of the recorded 3D/4D transperineal ultrasound volumes were performed by experienced investigators who were blinded against all clinical and obstetric data [17]. Analysis was done using specialized 3D imaging software, 4D View version 17.0 (GE Healthcare, Chalfont St. Giles, UK). The anterior-posterior (AP) diameter of the levator hiatus, left-right (LR) transverse diameter of the levator hiatus and area of the levator hiatus were measured at rest, PFMC and maximal Valsalva (Fig. 1). Relative changes in levator hiatal dimensions between rest and maximum Valsalva ([dimension at maximum Valsalva – dimension at rest/dimension at rest] × 100%) and between rest and PFMC ([dimension at contraction – dimension at rest/dimension at rest] × 100%) were computed [16].

### Statistical analysis

For this analysis we excluded women who were pregnant at the time of examination, as pregnancy has the potential to compromise sexual function and sexual activity [20, 21].

Measurements of hiatal dimensions followed a non-normal distribution. Linear regression analysis was used to compare measurements of hiatal dimensions and total PISQ-12 score. We used R^2^ to interpret the explained variance. Mann-Whitney U test was used to compare hiatal dimensions between women with and without individual sexual complaints. Baseline and obstetric factors were compared between women with and without sexual complaints using Student's t-test for continuous variables, Kendall’s tau-c for ordinal variables and chi-square/fisher’s exact test for categorical variables. Significant univariate associations were subjected to multivariate analysis using logistic regression analysis.

As this was a secondary analysis of the EpiLeva data, the sample size was determined by the primary end points of the EpiLeva study [17] and not specific to the research question addressed in this article. Statistical analysis was performed using IBM SPSS Statistics version 25. *P* values < 0.05 were considered statistically significant.

### Ethical approval

Ethical approval for this study was obtained from the medical research ethics committee of the Erasmus Medical Centre (MEC-2012-058). Date of approval was 11 April 2012.

## Results

A total of 2535 women were invited, and 204 (8%) agreed to participate in our study. Comparison of baseline characteristics between participants and non-participants was previously described [17]. In summary, participants were more likely to be Caucasian and had a longer second stage of labor. In non-participants more women were induced and more frequently received epidural analgesia. There were no significant differences in age, BMI, episiotomy rate and other obstetrical factors.

Thirteen (6%) women were pregnant at the time of examination and were excluded from the analysis. The median time at investigation after vaginal delivery was 11 months (range 6–33). The baseline and obstetric characteristics of the 191 included women are shown in Table 1.

**Table 1:** Baseline and obstetric characteristics of 191 included women in N(%)

Baseline		
	Age	30 (20–40)*
	Ethnicity: Caucasian	174 (91%)
	BMI at booking	24 (16–39)*
Obstetric		
	Perineal tear	
	Intact perineum	39 (21%)
	1st degree tear	16 (8%)
	2nd degree tear	38 (20%)
	Episiotomy	98 (51%)

*Mean (range)

### Sexual functioning and levator hiatal dimensions

All women had resumed sexual activity at the time of examination. Median (range) of the PISQ-12 was 38 (22–46). In our population 31 (16%) women experienced dyspareunia, 24 (13%) had difficulty reaching an orgasm, 33 (17%) experienced reduced desire, and 7 (4%) had a problem with arousal.

Using linear regression analysis we did not find a significant association between total PISQ-12 score and (relative changes in) levator hiatal dimensions, accept for AP diameter at rest (*p* = 0.014). Larger diameter is associated with higher PISQ-12 score and thus with better sexual functioning. However, this association is very weak, as < 5% of variance in the PISQ score is explained by the AP diameter at rest (R^2^ = 0.031).

Table 2 shows the comparison of hiatal dimensions between women with and without dyspareunia. These results indicate that women with dyspareunia had significantly smaller levator hiatal areas and AP diameter on maximum Valsalva. No significant effects were found for LR diameter. Table 3 shows the comparison in relative changes between levator hiatal dimensions between rest and PFMC and between rest and maximal Valsalva in women with and without dyspareunia. This showed no significant differences.

**Table 2:** Median (range) levator hiatal dimensions at rest, on pelvic floor muscle contraction and on maximal Valsalva in women with and without dyspareunia using Mann-Whitney U test

	Dyspareunia *N* = 31	No dyspareunia *N* = 160	*P* value
Rest			
Hiatal area	13.12 (7.84 – 20.53)	14.03 (8.18 – 34.13)	0.133
AP diameter (cm)	4.92 (3.01 – 6.06)	5.09 (3.67 – 7.22)	0.074
LR diameter (cm)	3.77 (3.09 – 5.80)	3.92 (2.66 – 6.09)	0.402
Contraction			
Hiatal area	10.72 (7.13 – 19.39)	10.81 (6.68 – 26.28)	0.149
AP diameter	4.21 (2.87 – 5.54)	4.20 (2.66 – 7.05)	0.328
LR diameter	3.73 (2.64 – 5.46)	3.62 (2.63 – 6.17)	0.450
Valsalva			
Hiatal area	16.13 (7.90 – 33.43)	18.79 (7.38 – 53.69)	0.035
AP diameter	5.25 (3.22 – 6.95)	5.86 (3.18 – 28.91)	0.006
LR diameter	4.26 (2.83 – 6.78)	4.23 (2.94 – 7.41)	0.467

**Table 3:** Relative changes [median (range)] in levator hiatal dimensions between rest and pelvic floor muscle contraction and between rest and maximal Valsalva in women with and without dyspareunia using Mann-Whitney U test

	Dyspareunia N = 31	No dyspareunia N = 160	*P* value
Contraction			
Hiatal area (%)	−16.05 (−44.22 – 5.55)	−18.63 (−55.18 – 69.49)	0.386
AP diameter (%)	− 13.69 (−32.54 – 11.25)	−15.78 (-44.12 – 45.66)	0.236
LR diameter (%)	−4.58 (−22.00 – 22.12)	−7.73 (-32.68 – 28.45)	0.149
Valsalva			
Hiatal area (%)	30.60 (−28.74 – 91.80)	31.87 (−29.78 – 197.87)	0.308
AP diameter (%)	10.25 (−19.04 – 61.79)	15.35 (−21.87 – 456.32)	0.230
LR diameter (%)	7.03 (−28.54 – 53.57)	9.43 (−21.66–63.29)	0.479

For the other individual sexual complaints there was no significant difference between levator hiatal dimensions and relative changes in levator hiatal dimensions. There were no significant differences between women with and without reduced desire, women with and without reduced arousal and women with and without difficulty reaching an orgasm.

### Multivariate analysis

We used multivariate analysis to correct for age, BMI at booking, ethnicity (Caucasian vs. non-Caucasian), postnatal follow-up time (weeks), grade of tear/episiotomy and other sexual complaints including reduced desire, reduced arousal and difficulty reaching an orgasm. Variables that showed a (near) significant association at univariate analysis were included in a multivariate model. In the final model for dyspareunia we included age, ethnicity, hiatus at maximal Valsalva, AP diameter at maximal Valsalva, the presence of reduced desire and reduced arousal. On multivariate analysis using logistic regression we found dyspareunia was independently significantly associated with younger age, reduced desire and reduced arousal, but not with hiatal dimensions at maximal Valsalva.

## Discussion

This study aimed to determine the association between levator hiatal dimensions and relative changes in levator hiatal dimensions and sexual dysfunction after first vaginal delivery. All women were sexually active at time of investigation. Sixteen percent of women experienced dyspareunia, 13% had difficulty reaching an orgasm, 17% experienced reduced desire, and 4% had a problem with arousal. Postpartum sexual dysfunction (including dyspareunia) is identified in 41–83% of women at 2–3 months postpartum [1]. Longer term data beyond 6 months postpartum are sparse. There is great variability in the literature on the reported prevalence rates of particular sexual dysfunctions due to differences in the definition of sexual dysfunction, degree of severity and differences in age groups. Similar to our data, prevalence rates for women in the general population vary from 17% to 55% for low levels of sexual desire, from 8% to 28% for arousal and lubrication problems, from 16% to 25% for orgasmic dysfunction and from 14% to 27% for dyspareunia [22].

We hypothesized that women that experience sexual complaints show significant differences in levator hiatal dimensions and relative changes in these dimensions on PMFC and maximal Valsalva compared with women without sexual complaints. However, our results showed that 11 months after first vaginal delivery hiatal dimensions are not predictive of overall sexual dysfunction as measured by PISQ-12. When we studied individual sexual complaints we found that dyspareunia was associated with smaller hiatal dimensions at Valsalva on univariate analysis, but on multivariate analysis, hiatal dimensions were not an independent risk factor for dyspareunia.

Previous studies showed that at 5 months after delivery, levator ani muscle avulsion and levator over-distension did not have appreciable effects on women’s sexual functioning related to sexual activity, sensation on intercourse, arousal and orgasm [14]. Although women with persistent levator avulsion had significantly more bothersome symptoms of a loose vagina at 1 year postpartum, these symptoms had no impact on their sexual life [15]. Although levator avulsion was associated with larger hiatal areas on transperineal ultrasound in these studies, the association between the different levator hiatal dimensions and sexual function was not examined. A study by Aydin et al. did correlate levator hiatal dimensions and sexual dysfunction in non-symptomatic sexually active premenopausal women [23]. An increase in AP diameter of the levator hiatus during maximal Valsalva was weakly associated with worse sexual functions, particularly desire, arousal and orgasm domains [23]. Our study demonstrates that after first vaginal delivery hiatal dimensions were not associated with arousal, desire or orgasm. Although not significant on multivariate analysis, we did find that women with dyspareunia had smaller hiatal dimensions at Valsalva rather than larger dimensions, which could be a sign of higher pelvic floor muscle tone.

The etiology of sexual dysfunction is multifactorial, including emotional, physical and psychological causes. Changes in the dimensions and contractility of the pelvic floor might be the main cause of sexual dysfunction only in a subgroup of women. Perineal pain resulting from perineal trauma, fear of pain on resumption of intercourse or psychological stress associated with changes in family structure following childbirth could possibly cause an increase in pelvic floor muscle tone in the postpartum period. Previous studies have shown that levator hiatal dimensions are smaller in women diagnosed with provoked vestibulodynia (PVD). In a study by Thibault et al., women were diagnosed with PVD when complaining about pain around the vaginal opening upon attempted vaginal penetration for a minimum of 6 months [16]. The diagnosis was confirmed by using a standardized cotton-swab test. Women with PVD had smaller hiatal dimensions at rest, on PFMC and on maximum Valsalva than controls. Furthermore, these women had smaller relative increases in hiatal area on maximum Valsalva, which is associated with hypertonia from the pelvic floor muscles.

When dyspareunia is caused by, or maintained as a result of, pelvic floor hypertonia without anatomical causes, first-line treatment consists of education, tactile desensitization and pelvic floor physiotherapy possibly in combination with psychological or sexological consultation. When first-line treatment fails, more invasive interventions can be considered. One of these invasive interventions is injection with botulinum toxin A (BTA) in the pelvic floor muscles. When injected intramuscularly BTA produces a localized, partial and reversible chemical denervation of the muscle, which results in localized muscle weakness or paralysis. There is some low-grade evidence that injection of BTA in the hypertonic pelvic floor muscles decreases pelvic floor resting pressure as measured by manometry and improves dyspareunia in women with therapy-resistant chronic pelvic pain [24, 25]. Pelvic floor ultrasound might play a role in the future in the diagnostic workup of women with dyspareunia. A cutoff value for high muscle tone needs to be determined. These women with high muscle tone could be a subgroup of women with dyspareunia that might benefit from BTA injections.

To the best of our knowledge, this is the first study to evaluate the association of sexual complaints and the biometry of the levator hiatus and relative changes of the puborectalis muscle using 3D/4D transperineal ultrasound in the postpartum period. All women in our study filled out the validated PISQ-12 questionnaire. The women included in our study did not have an increased risk for sexual dysfunction postpartum, as women who had instrumental delivery or third- or forth degree perineal tearing were excluded [1]. Furthermore, the researchers who conducted the study were blinded against all clinical and obstetric data during analysis of the ultrasound data, thereby reducing bias.

There were also some limitations to our study. First, there was a possibility of selection bias. Only 8% of invited women participated in our study. Lack of time and fear of the examination were the most mentioned reasons for non-participation. This might have led to a higher participation rate of women with complaints or complications. However, the percentage of women reporting sexual complaints in our study does not exceed the incidence reported in previous literature, suggesting a representative cohort [22]. Participants were more likely to be Caucasian, but did not differ in age and BMI compared with non-participants. We therefore believe that our results can be generalized to other populations of mainly Caucasian women who had a first spontaneous vaginal delivery at term. A second limitation is our sample size. As this was a secondary analysis of the EpiLeva data, the sample size was determined by the primary end points of the EpiLeva study and not specific to the research question addressed in this article [17]. It might be that our sample size was too small to evaluate a significant association between the biometry of the levator hiatus and sexual complaints. A third limitation is the fact that we did not ask about sexual functioning before and during pregnancy. However, due to the design of our study, it was impossible to prospectively collect these data as it would have required inclusion of study participants at the first antenatal booking. When information on pre-pregnancy sexual functioning was obtained retrospectively in the postpartum period, it might have been subject to recall bias. Finally, the PISQ-12 questionnaire may not be the best questionnaire to identify sexual dysfunction in our cohort of postpartum women. The PISQ-12 is validated to assess sexual function in women with urinary incontinence and/or pelvic organ prolapse, but has previously also been used to evaluate sexual function after vaginal delivery [26, 27]. The EpiLeva study was conducted to assess the association of mediolateral episiotomy with levator injury and urogynecological complaints. This is why we chose to use the PISQ-12 questionnaire. However, the frequency of pelvic organ prolapse and urinary incontinence symptoms in our study population was low. As the PISQ-12 does not provide cutoff values discriminating between normal sexual function and sexual dysfunction, we dichotomized answers of questions to what we considered to be relevant complaints. For future research on this topic a more general female sexual function questionnaire could be considered, for example, the Female Sexual Function Index (FSFI).

In conclusion, we can state that sexual dysfunction at 11 months postpartum is not independently associated with levator hiatal dimensions or relative changes in levator hiatal dimensions as measured by 3D/4D transperineal ultrasound. Although women with dyspareunia had smaller hiatal dimensions at maximum Valsalva, suggesting higher pelvic floor muscle tone, on multivariate analysis this association was not significant. Sexual dysfunction in the postpartum period is multifactorial and can have many different psychological as well as physical causes. Changes in hiatal dimensions of the pelvic floor could be one of the causes of sexual dysfunction in a certain group, but further research is necessary to identify these women.
